# Stepping up in clinical governance: The leadership challenge for new family physicians

**DOI:** 10.4102/safp.v67i1.6143

**Published:** 2025-04-10

**Authors:** Klaus B. von Pressentin, Arun Nair, Chantelle van der Bijl

**Affiliations:** 1 Department of Family, Community and Emergency Care, Faculty of Health Sciences, University of Cape Town, Cape Town, South Africa; 2 Department of Family Medicine, Faculty of Health Sciences, University of the Free State, Bloemfontein, South Africa; 3 Robert Mangaliso Sobukwe Hospital, Northern Cape Department of Health, Kimberley, South Africa

The first 5 years after qualification can be both exhilarating and overwhelming. As a newly minted family physician (FP), you have earned your spot in the health system, but the real challenge begins now. You are not merely a clinician but a leader in clinical governance tasked with improving health services. You might be wondering how to develop these essential skills. Let us explore the why, what and how of clinical governance leadership for early-career FPs.

## Why clinical governance?

South Africa’s healthcare is at a turning point. The push for universal health coverage and national health insurance requires stronger primary health care systems. Clinical governance aims to support and develop the capacity for implementing tools and systems that provide quality clinical services.^1^ However, recent reports have highlighted a lack of knowledge regarding clinical governance tools among clinical staff and the urgent need to develop training programmes for clinical managers.^2,3^ Without effective leadership, quality declines, inefficiencies grow, and patients suffer. Given that clinical governance is central to our postgraduate curriculum, FPs play a specific role in driving changes from within the system.^4,5^

## The pillars of clinical governance

An effective clinical governance leader engages with these fundamental aspects:

- *Quality improvement*: Using data-driven methods to enhance patient care by refining referral processes, minimising unnecessary hospitalisations, and improving chronic disease management.
- *Risk management and patient safety*: Conducting morbidity and mortality (M&M) meetings to foster a culture of learning from mistakes and ensure compliance with safety protocols.
- *Clinical effectiveness*: Grounding care in current evidence by applying clinical guidelines relevant to your health service.
- *Workforce development*: Supporting the learning and well-being of your multidisciplinary team, including mentoring junior professionals.
- *Resource stewardship*: Ensuring fair use of healthcare resources like staff and equipment, especially in resource-limited settings.
- *Patient and community engagement*: Promoting patient input in service design to make primary care accessible and responsive to community needs.

## How to develop clinical governance leadership

Entering clinical governance leadership is a gradual process rather than an instant transition. It demands deliberate efforts, but there are effective strategies to accelerate your development:

- *Embrace your leadership role*: Many newly qualified FPs hesitate to take the lead, waiting for permission or seniority. Avoid adopting this mindset; leadership depends on influence and relationships, not titles. Start developing and demonstrating your leadership skills in your clinical environment.
- *Develop relationships*: Leadership in clinical governance extends beyond patient interactions; it also involves influencing governance systems, the so-called ‘behind the scenes’ or non-patient-facing activities. Cultivate robust relationships with clinical and management team members, as this social capital is vital for securing support when engaging in quality improvement activities.
- *Identify clinical governance areas*: It is unrealistic for every FP to engage in all aspects of clinical governance simultaneously. Choose an area to influence, such as antimicrobial stewardship or maternal health audits. Develop expertise before expanding your focus.
- *Master quality improvement techniques*: Familiarise yourself with tools such as the Plan-Do-Study-Act (PDSA) cycle and root cause analysis. Use data instead of anecdotes to drive change. A range of modes of thinking will assist with tackling healthcare system challenges and opportunities.^6^
- *Lead essential meetings*: Participate in M&M meetings, governance committees and service enhancement discussions. Take initiative and lead key areas. Ensure follow-up on action items.
- *Acquire leadership training*: Leadership demands skills such as negotiation, conflict resolution and supporting colleagues and junior staff facing difficulties. Consider seeking mentorship from experienced FPs. Use resources like the South African Family Practice Manual and the World Health Organization’s Quality Improvement Toolkit.^7,8^
- *Learn from mistakes and evolve*: Not every initiative will succeed. Reflect, learn and adapt your approach. Clinical governance is a long-term endeavour that requires resilience and a learning health system mindset. A community of practice will ensure a constructive learning culture with colleagues at different career stages, including students and registrars.

## Real-world impact: Stories of family physician-led change

A recent special collection highlighted FPs’ role in strengthening health systems through clinical governance.^9^ Across South Africa, family physicians have demonstrated how clinical governance leadership transforms services. Some examples include:

- *Expanding access to key primary health care services*: Over time, a multidisciplinary team developed services, including antibiotic stewardship, palliative care, staff training and a smoking cessation clinic for staff.
- *Safely switching antiretroviral regimens*: In 2019, the coronavirus disease 2019 (COVID-19) pandemic disrupted services, prompting primary care doctors to launch a project facilitated by their FP to safeguard renal function in patients initiating or switching to dolutegravir-based antiretroviral therapy.
- *Digital technologies for health*: FPs innovated with technology for health information and service delivery linked to data management and research.
- *Care coordination between levels of care*: A district-level referral forum between levels of care improved patient flow, improved access to specialist input and significantly reduced the number of referrals.

## Next steps for you to explore

Transitioning from registrar to FP is an exciting journey fuelled by mentorship and self-reflection. We invite you to join the South African Academy of Family Physicians (SAAFP)’s Next5 mentorship programme, where you can cultivate your growth as a leader and make a significant impact as a new FP.^10^ Leadership is a meaningful journey; start small and remain consistent.
